# Pyrroloquinoline quinone modulates YAP-related anti-ferroptotic activity to protect against myocardial hypertrophy

**DOI:** 10.3389/fphar.2022.977385

**Published:** 2022-09-27

**Authors:** Jiabin Zhou, Tao Yu, Gujie Wu, Peng Xu, Chen Wang, Yiling Su, Li Wang, Qi Lu

**Affiliations:** ^1^ Department of Cardiology, Affiliated Hospital of Nantong University, Nantong, China; ^2^ Medical School, Nantong University, Nantong, China; ^3^ Department of Cardiovascular Surgery, Affiliated Hospital of Nantong University, Nantong, China

**Keywords:** pyrroloquinoline quinone, yes-associated protein, lipid peroxidation, ferroptosis, myocardial hypertrophy

## Abstract

**Background:** Pyrroloquinoline quinone (PQQ) has been reported to exhibit cardioprotective and antioxidant activities. Accordingly, this study was developed to explore the effects of PQQ treatment on myocardial hypertrophy and the underlying mechanism of action governing any observed beneficial effects.

**Methods:** A transverse aortic constriction (TAC) model of myocardial hypertrophy was established *in vivo* using C57BL/6 mice, while neonatal murine cardiomyocytes were stimulated with phenylephrine (PE) as an *in vitro* validation model system.

**Results:** Treatment of TAC model mice with PQQ significantly suppressed myocardial hypertrophy and fibrosis, in addition to inhibiting the ferroptotic death of hypertrophic myocardial cells *in vivo.* Subsequent *in vitro* analyses revealed that treatment with PQQ was sufficient to significantly alleviate PE-induced hypertrophic activity and to prevent ferroptotic induction in these primary murine cardiomyocytes. At the mechanistic level, PQQ was found to promote the upregulation of Yes-associated Protein (YAP), to suppress YAP phosphorylation, and to drive the nuclear translocation of YAP within hypertrophic cardiomyocytes. The use of a specific siRNA construct to knock down YAP expression *in vitro* further confirmed the ability of PQQ to protect against myocardial hypertrophy at least in part through anti-ferroptotic mechanisms.

**Conclusion:** PQQ can regulate the pathogenesis of myocardial hypertrophy through the induction of YAP-related anti-ferroptotic activity, highlighting the potential value of PQQ as a novel therapeutic agent capable of slowing or preventing the progression of myocardial hypertrophy and thus delaying the onset of heart failure.

## Introduction

Myocardial hypertrophy is an adaptive response of the cardiac tissue to injury or certain other stimuli, resulting in hypertrophic activity, cell death, interstitial fibrosis, and the consequent disruption of normal myocardial cell structures (Latunde-Dada, 2017). Associated compensatory activity cannot be maintained indefinitely such that pathological myocardial hypertrophy can eventually cause heart failure. At present, therapeutic options for the treatment of myocardial hypertrophy are limited, underscoring the need for the design or discovery of novel drugs capable of treating this condition or slowing its progression to improve patient outcomes.

Ferroptosis is a form of iron-dependent programmed cell death associated with extensive lipid peroxidation. Ferroptotic cells exhibit characteristic features including outer mitochondrial membrane rupture, unusually small mitochondria, and less pronounced or even absent cristae (Mou et al., 2019). Excessive intracellular levels of iron ions generate hydroxyl radicals through the Fenton reaction interacting with hydrogen peroxide, thus promoting the production of reactive oxygen species (ROS), which cause lipid peroxidation and are believed to be the primary driver of ferroptotic cell death (Su et al., 2019), and this peroxidative damage can be further exacerbated by reductions in the activity of glutathione peroxidase 4 (GPX4) and glutathione (GSH) together with enhanced malondialdehyde (MDA) activity (Conrad and Sato, 2012; Ayala et al., 2014; Ingold et al., 2018). In contrast, ferroptosis suppressor protein 1 (FSP1, also known as AIFM2) can potently inhibit this form of cell death (Doll et al., 2019) by inducing coenzyme Q10 (CoQ10) production and thereby suppressing lipid peroxidation (Bersuker et al., 2019).

Yes-associated protein (YAP) is a downstream transcriptional activator in the Hippo pathway that controls the survival and proliferation of cardiomyocytes and many other cells, regulating a diverse array of oxidative stress- and apoptosis-related pathological and physiological activities (Ramos and Camargo, 2012). YAP can mediate pressure overload-related adaptive cardiac hypertrophy (Byun et al., 2019), and exposure to oxidative stress can promote the phosphorylation of YAP, contributing to the exacerbation of myocardial ischemia/reperfusion injury (Matsuda et al., 2016). YAP has also recently been shown to serve as a regulator of ferroptotic activity (Yang and Chi, 2020), promoting breast cancer cell ferroptosis through the E3 ligase Skp2 (Yang et al., 2021). Moreover, cell-cell interactions have been shown to contribute to the induction of mesenchymal carcinoma cell ferroptosis through mechanisms dependent on NF2-YAP signaling (Wu et al., 2019). However, the specific role that YAP plays in the context of ferroptotic cell death in the context of pathological cardiac hypertrophy remains to be established.

Pyrroloquinoline quinone (PQQ) is a natural water-soluble redox coenzyme that is found throughout plant and animal tissues, serving as an essential nutrient in mammals with purported cardioprotective activity (Akagawa et al., 2016). Moreover, PQQ has been shown to exhibit antioxidant activity in a rat model of diabetic nephropathy (Zhang et al., 2020), and to protect against diabetic cardiomyopathy via the inhibition of scorched death signaling activity (Qu et al., 2022). Nehra et al. (2017) further developed a PQQ- and curcumin-containing nano-curcumin formulation capable of reducing the severity of hypoxia-induced ventricular myocyte hypertrophy and injury.

In light of the above evidence, the present study was developed with the hypothesis that PQQ may act as a cardioprotective agent to prevent heart failure via modulating YAP-related anti-ferroptotic activity, thus reducing the severity of myocardial hypertrophy. Together, the results of this study have the potential to offer new insight into the mechanisms governing YAP signaling and ferroptosis, providing promising new clinical directions for the treatment of heart failure patients.

## Materials and methods

### Reagents

PQQ was obtained in the form of a disodium salt from Nascent Health Sciences LCC (NY, USA). Phenylephrine (PE) was from Sigma-Aldrich (MO, USA). Primary antibodies specific for YAP, GPX4, FSP1, and CoQ10 were from ABclonal (A1002, A1933, A12128, and A15193), while antibodies specific for p-YAP, BNP, β-actin, GAPDH, and Histone H3, as well as secondary goat anti-rabbit and goat anti-mouse IgG were from Wuhan Servicebio Technology Co., Ltd. (GB114060, GB11667, GB12001, GB15002, GB11026, GB23303, and GB23301). The 2′,7′-dichlorofluorescein diacetate (DCFH-DA) (S0033) and the mitochondrial membrane potential assay kit JC-1 (C2006) were purchased from the Beyotime Institute of Biotechnology. A test kit for measuring ferrous ion concentration (E-BC-K773-M) was purchased from Elabscience (Wuhan, China), and the kits for measuring malondialdehyde (MDA) and glutathione (GSH) levels were obtained from Nanjing Jiancheng Institute of Biological Engineering (A003-4-1, A006-2–1). Ferrostatin-1(Fer-1) was purchased from Selleck Chemicals (USA). Gibco (CA, United States) was the source of all cell culture reagents.

### Animal experiments

Specific pathogen-free C57BL/6 mice (male, 8-weeks-old, 20–25 g) were obtained from the Experimental Animal Center of Nantong University and housed with free food and water access in a climate-controlled facility (21 ± 1°C, 55%–60% humidity). The Animal Ethics Committee of Nantong University approved all animal studies, which were consistent with the Guide for the Care and Use of Laboratory Animals. Mice were randomized into sham surgery + PBS, sham surgery + PQQ, TAC surgery + PBS, and TAC surgery + PQQ treatment groups (n = 6/group). A TAC-induced pressure overload model was established as in prior reports. Mice were observed for 6 h after surgery, and were then transferred to an experimental animal center for standardized feeding. Sham-operated control mice had sutures passed through the soft tissue under the aortic arch, but no ligation was performed. All other steps were the same as for TAC-operated mice. Following surgery, mice were monitored daily and had free food and water access.

PQQ (at a dose equivalent to previous studies (Qu et al., 2022); 40 mg/kg in PBS) or an equal volume of PBS were administered to mice *via* gavage every other day for a 6-week period beginning on day 2 post-surgery. The survival rate of mice after TAC remained above 80% among all groups and did not differ significantly. At 6 weeks post-surgery, surviving mice underwent echocardiographic evaluation, with surviving animals then being used for other assays as appropriate.

### Transthoracic echocardiography

Mice were initially anesthetized using isoflurane, fixed on the operating table in a supine position, followed by the preparation of the skin in the anterior thoracic region which was coated with a small volume of coupling agent. Echocardiography was performed with a high-resolution small animal ultrasound imaging system (Vevo 2,100), probe FMS-250, adjusted to a depth of 2.0–2.5 cm and a frequency of 13–24 MHz. During M-mode ultrasonographic visualization of cardiac structures, the left ventricular internal diastolic diameter (LVIDd), left ventricular internal diameter end-systole (LVIDs), and interventricular septal dimension in diastole (IVSd) were measured in parasternal left ventricular long-axis and left ventricular short-axis views. These values were then processed to calculate indices related to left ventricular systolic function including ejection fraction (EF) and fractional shortening (FS), with all parameters being reported as the average from 3-5 cardiac cycles.

### Histological analyses

Murine cardiac tissue samples were isolated, fixed for 48 h with 4% paraformaldehyde, paraffin-embedded, and cut into 5 mm cross-sections that were stained with hematoxylin and eosin (H&E) to assess histopathological changes. In addition, myocardial cell cross-sectional area was measured via WGA staining, while Masson’s trichrome staining was used to assess the degree of interstitial fibrosis in the murine myocardium. The NIH ImageJ software was used to assess cell area and fibrotic area.

### Cell culture

Murine HL-1 and H9C2 cardiomyocytes were purchased from the Shanghai Institute of Biological Sciences, Chinese Academy of Sciences, and were cultured in high-glucose DMEM containing 10% FBS and penicillin/streptomycin at 37°C in a 5% CO_2_ incubator.

To isolate cardiac myocyte from C57BL/6 mice, cardiac tissue samples were digested using collagenase and maintained in media containing 10% FBS and penicillin/streptomycin, with cardiac myocytes being purified via differential applanation. Cardiac fibroblasts were excluded using 0.1 mmol/L BrdU (5-bromo-2-deoxyuridine). Following plating, cells were incubated for 48 h. Hypertrophy was induced by treating cardiac myocyte with PE (50 μM) for 48 h, followed by incubation with or without PQQ (100 μM) for an additional 24 h.

Lipofectamine 3,000 (Invitrogen, CA, United States) was used to transfect cells with YAP-specific siRNA (5′-GGG​UAA​GUC​GAG​AAG​UGU​UTT-3′) or control (si-NC) constructs from GenePharma (Shanghai, China). Knockdown efficiency was confirmed at 48 h post-transfection.

### Cell viability assays

Cell survival was assessed via CCK-8 assay. Briefly, cells were added to 96-well plates (5 × 10^3^)/well and incubated in the presence of a range of PQQ concentrations (1, 5, 10, 25, 50, 100, 150, 200 µM), with six replicate samples per condition. Then the CCK-8 reagent was added to each well (10µL/well), followed by incubation for an additional 2 h. Absorbance at 450 nm was then assessed. Alternatively, after culture for 24 h, cells were similarly treated with a range of PE concentrations (1, 5, 10, 25, 50, 100, 150, 200 µM), with six replicates per sample. Viability was then assessed at 48 h post-treatment with a CCK-8 assay kit (Beyotime, Shanghai, China) as above. Survival was normalized to that of control untreated cardiomyocytes (100%).

### Cell surface area measurements

Primary murine cardiomyocytes were fixed for 20 min with 4% paraformaldehyde and sealed for 2 h using QuickBlock Immunostaining Closure Solution (Beyotime, Shanghai, China) at room temperature. Samples were then incubated overnight with anti-α smooth muscle actin (Abcam, MA, United States; ab124964) at 4 °C followed by incubation for 2 h with secondary goat anti-rabbit IgG H&L (Alexa Fluor^®^ 488; Abcam; ab150081) in the dark. Cells were then imaged via fluorescent microscopy, with cell surface area being quantified with the NIH ImageJ software application.

### qPCR

Trizol (Servicebio, Wuhan, China) was used to extract total RNA from cardiac tissue and neonatal murine cardiomyocytes, after which cDNA was prepared with HiScript II Q RT SuperMix (Vazyme, Nanjing, China), and qPCR was performed with the ChamQ universal SYBR qPCR Master Mix (Vazyme, Nanjing, China) and a fluorescent quantitative PCR instrument (Bio-rad; CFX). Primer sequences used were as follows:

GAPDH (F: CCT​CGT​CCC​GTA​GAC​AAA​ATG, R: TGAGGTCAATGAAGGGGTCGT);ANP (F: CCG​ATA​GAT​CTG​CCC​TCT​TGA​A, R: GCTGTTGCAGCCTAGTCCACT);BNP (F: GGA​GGC​GAG​ACA​AGG​GAG​AA, R: CCAGCGGTGACAGATAAAGGAA);β-MHC (F: ATG​AGG​AGT​AGC​TCT​TGT​GCT​ACC, R: CCACCTAAAGGGCTGTTGC);All primers were synthesized by RiboBio (Guangzhou, China). GAPDH served as a normalization control. Relative gene expression was assessed via the 2^−ΔΔCT^ method.

### Western immunoblotting

A protein extraction reagent was used to isolate proteins from tissue or cell samples, after which a BCA protein analysis kit (Servicebio, Wuhan, China) was used to measure protein concentrations. Proteins were then separated via 8% SDS-PAGE (Servicebio, Wuhan, China), transferred to PVDF membranes, and blots were then blocked for 2 h at room temperature with 5% non-fat milk prior to incubation overnight with appropriate primary antibodies at 4°C. Blots were rinsed with TBST three times, then probed with secondary antibodies for 2 h. Chemiluminescent reagents were then used to detect protein bands, followed by quantification using ImageJ.

### Transmission electron microscopy

After collection, cardiac tissue samples were fixed for 10 min with 1% glutaraldehyde, followed by further fixation for 1 h at room temperature using 3% glutaraldehyde. Samples were then further fixed for an additional 1 h at room temperature using 1% osmium tetroxide in 0.1 mol/L dimethylarsinate buffer, followed by embedding in resit. Polymerized ultrathin sections were then stained using uranyl acetate and lead nitrate, followed by TEM imaging to assess mitochondrial morphology and transverse muscle arrangement.

### Mitochondrial membrane potential analysis

The fluorescent JC-1 probe was used to measure MMP (ΔψVm). Briefly, cardiomyocytes were treated with PQQ as above and were then incubated for 20 min with a JC-1 working solution at 37 °C, followed by two washed using JC-1 staining buffer. Monomeric and polymeric JC-1 exhibited excitation maxima of 514 and 585 nm, respectively. A fluorescent microscopy was then used to analyze cells to detect JC-1 fluorescence.

### ROS detection and quantification

DCFH-DA staining was used to assess ROS production. Briefly, cells treated as above were added to 24-well plates and incubated for 20 min with DCFH-DA (10 μmol/L) at 37 °C. Cells were then rinsed thrice using serum-free culture media to eliminate any extracellular DCFH-DA, and cells were imaged via fluorescent microscopy (Ex: 488 nm, Em: 522 nm) to assess intracellular ROS levels.

### Analyses of MDA and GSH levels

After cells had been harvested and lysed, a BCA kit was used to measure protein concentrations. MDA levels in these cells were assessed using an MDA assay kit, with absorbance at 530 nm ultimately being measured. Similarly, GSH levels in these cells were analyzed based on the directions provided with a micro-reduced GSH assay kit. Absorbance was assessed at 405 nm, and GSH levels were established based on a standard curve.

### Iron ion quantification

Cells were treated as above, and a BCA kit was used to detect protein concentrations in sample lysates. A ferrous ion colorimetric test kit was then used to detect iron ion levels based on provided directions. Briefly, the provided probe bound to ferrous ions released by cells, with the resultant absorbance at 593 nm being quantified to measure intracellular Fe2^+^ content.

### Statistical analysis

Data are reported as means ± SEM, and were analyzed using GraphPad Prism 9.3.0. Differences between groups were compared *via* Student’s *t*-tests or one-way ANOVAs with Bonferroni post hoc tests. *p* < 0.05 was the significance threshold.

## Results

### PQQ protects against myocardial hypertrophy and fibrosis in a TAC-induced mouse model system

**FIGURE 1:**
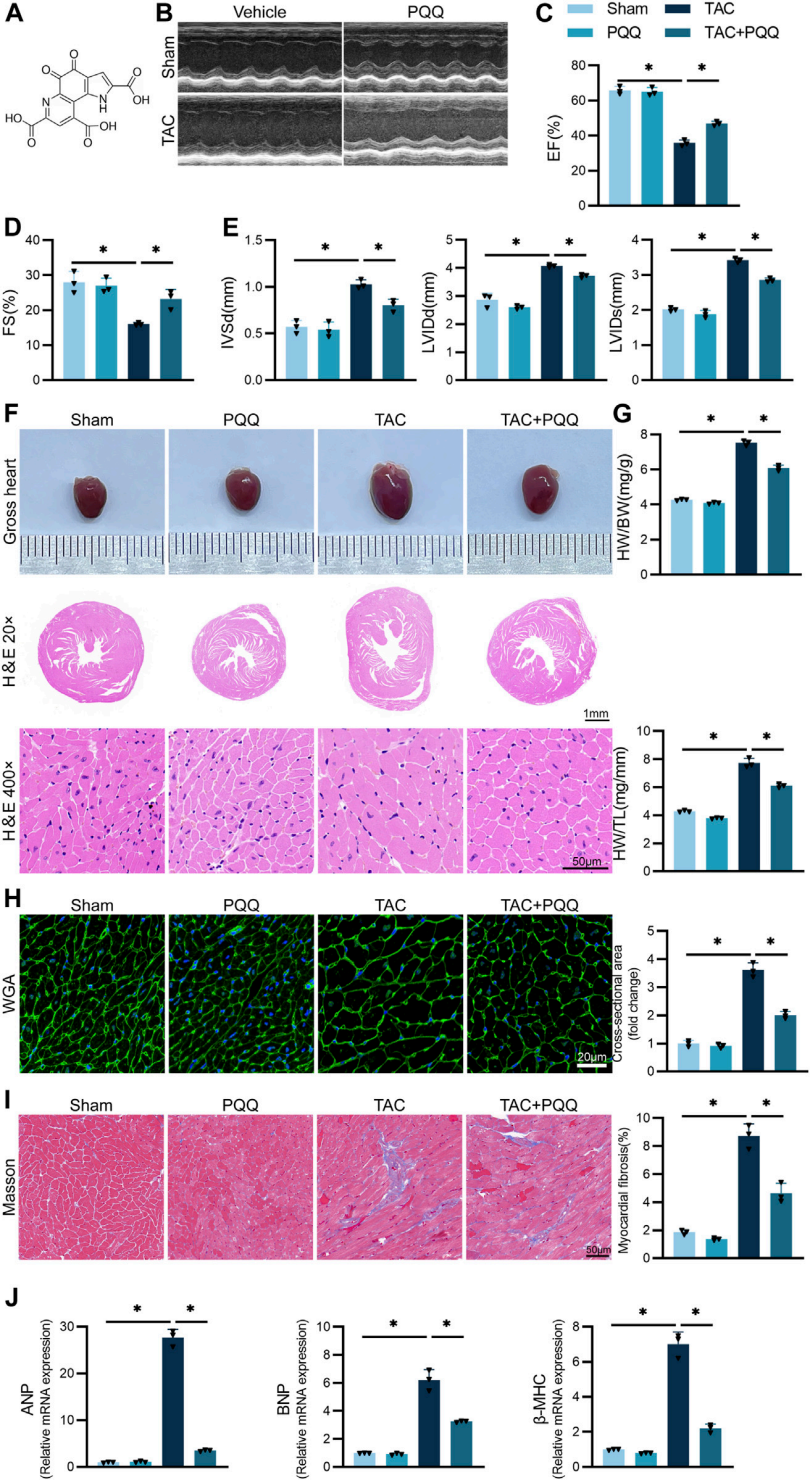
PQQ administration attenuated TAC-induced cardiac hypertrophy and fibrosis in a mouse model system **(A)** Thehemical structure of PQQ. **(B–E)** Mice in the indicated groups underwent M-mode echocardiographic imaging to assess left ventricle parameters in the long axis of the left parasternal sternum. Analyzed parameters included ejection fraction (EF), fractional shortening (FS), left ventricular internal diastolic diameter (LVIDd), left ventricular internal diameter end-systole (LVIDs), and interventricular septal dimension in diastole (IVSd). **(F)** Representative of gross cardiac morphology and H&E-stained cross-sections (40x and 200x) assessing cardiomyocyte morphology. **(G)** Quantification of heart weight (HW) relative to body weight (BW) or tibial length (TL) in the indicated mice. **(H)** WGA staining and cell surface area quantification-based analyses of cardiomyocyte cross-sectional area (CSA). **(I)** Cardiomyocyte fibrosis was detected and quantified via Masson’s trichrome staining. **(J)** The relative expression of the hypertrophy biomarkers ANP, BNP, and β-MHC was assessed via qPCR. Data are means ± SEM. *p < 0.05. N = 3/group. Data were compared via one-way ANOVAs with Bonferroni post hoc tests.

Initially, a C57BL/6 mouse model of myocardial hypertrophy was established by using previously published protocols to perform the TAC procedure, with a subset of these mice being treated using PQQ (Figure 1A). At 6 weeks post-TAC surgery, echocardiography analyses of these mice revealed that TAC-operated animals exhibited significant reductions in EF and FS relative to sham control mice, whereas IVSd, LVIDd and LVIDs were increased, while PQQ treatment partially reversed this TAC-induced cardiac dysfunction (Figures 1B–E). TAC-operated mice also exhibited other features consistent with cardiac hypertrophy, including significantly increased heart volume (Figure 1F), as well as significant increases in heart weight relative to body weight or tibial length (Figure 1G). H&E and WGA staining also revealed that the cardiomyocyte cross-sectional area in the TAC group was significantly increased, whereas PQQ treatment reversed this change (Figures 1F,H). Masson’s trichrome staining additionally indicated that PQQ-treatment protected against TAC-induced myocardial fibrosis in these animals (Figure 1I). TAC surgery was associated with the upregulation of the hypertrophic cardiomyocyte biomarkers atrial natriuretic peptide (ANP), brain natriuretic peptide (BNP), and β-myosin heavy chain (β-MHC), whereas these levels were significantly decreased in cardiac tissue samples from PQQ-treated mice that underwent TAC surgery (Figure 1J). Together, these data suggested that PQQ treatment was sufficient to protect mice against cardiac hypertrophy and fibrosis induced in response to the TAC procedure.

### PQQ administration prevents the *in vivo* induction of ferroptotic cell death in hypertrophic myocardial tissue

**FIGURE 2:**
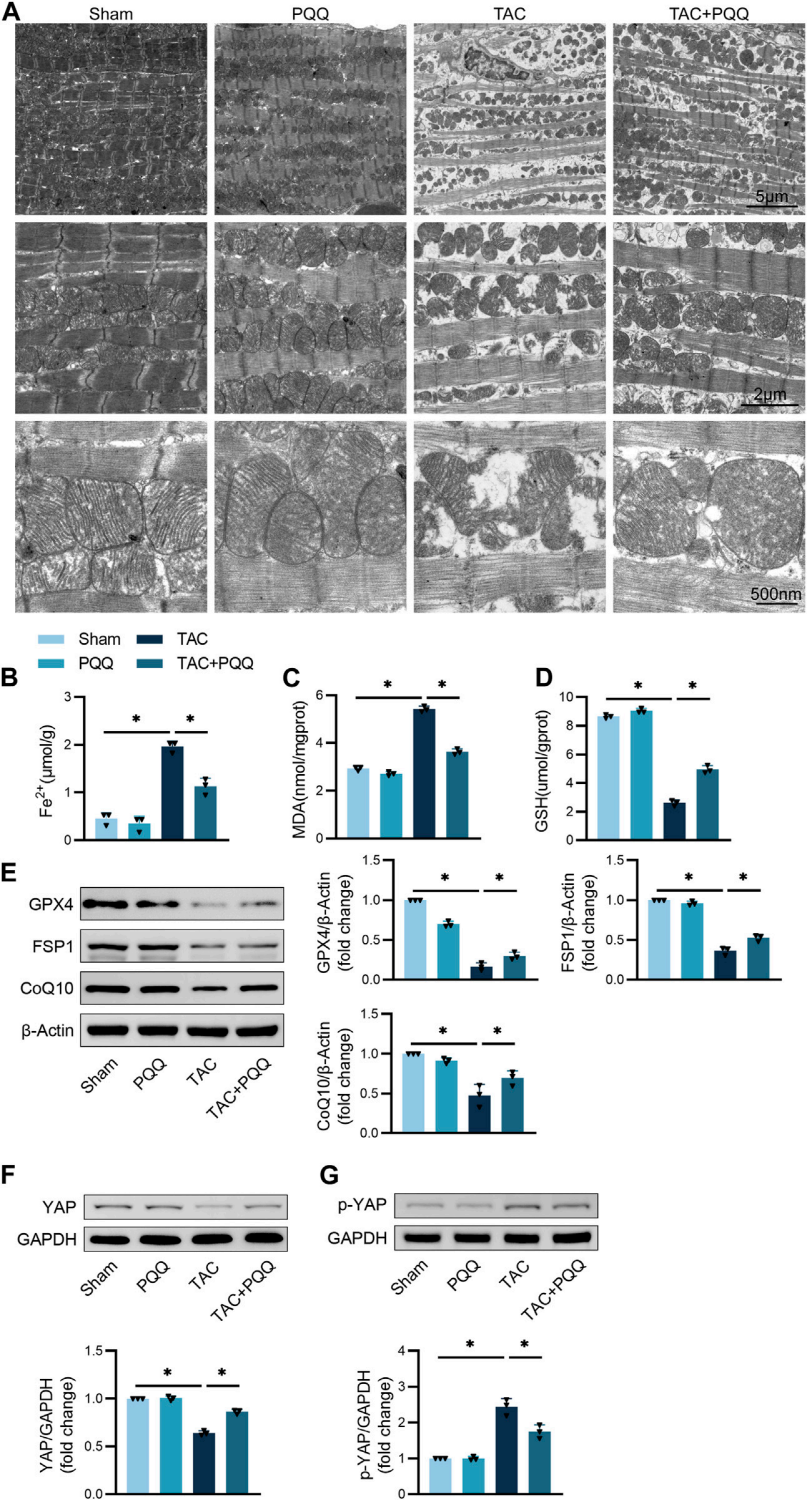
The *in vivo* administration of PQQ inhibits the ferroptotic death of hypertrophic cardiomyocytes. **(A)** Representative images of the ultrastructural morphological characteristics of cardiomyocyte mitochondria. **(B–D)** Iron **(B)**, MDA **(C)**, and GSH **(D)** levels in murine cardiomyocytes were quantified with appropriate commercial kits. **(E)** Representative Western immunoblots with corresponding quantification assessing GPX4, FSP1, and CoQ10 levels in primary cardiomyocytes. **(F)** Representative Western immunoblots with corresponding quantification assessing YAP levels in primary cardiomyocytes. **(G)** Representative Western immunoblots with corresponding quantification assessing p-YAP levels in primary cardiomyocytes. Data are means ± SEM. *p < 0.05. N = 6/group. Data were compared via one-way ANOVAs with Bonferroni post hoc tests.

Next, the morphological characteristics of mitochondria in cardiomyocytes from these different murine treatment groups were assessed via TEM. TAC surgery was associated with mitochondrial that were smaller and more disordered, with outer membrane rupture and cristae that were absent or reduced. PQQ treatment of TAC-operated mice, however, was sufficient to significantly reduce the extent of mitochondrial outer membrane rupture and to increase the number of visible mitochondrial cristae (Figure 2A). Iron ion and MDA levels in the cardiac tissue of TAC-operated mice were significantly increased, while PQQ treatment reversed these changes (Figures 2B,C). Conversely, GSH levels declined following TAC treatment but were restored in PQQ-treated mice (Figure 2D). Western immunoblotting analyses indicated that the anti-ferroptotic proteins Gpx4, FSP1, and CoQ10 were upregulated in the TAC + PQQ group relative to the TAC group (Figure 2E). Lastly, YAP and p-YAP levels were assessed in these murine cardiac tissue samples, revealing higher p-YAP and reduced YAP levels in TAC-operated mice, whereas PQQ administration partially reversed these changes (Figures 2F,G). These results thus underscored the potential for further research aimed at clarifying the mechanisms underlying these phenotypes.

### PQQ protects against PE-induced cardiomyocyte hypertrophy *in vitro*

**FIGURE 3:**
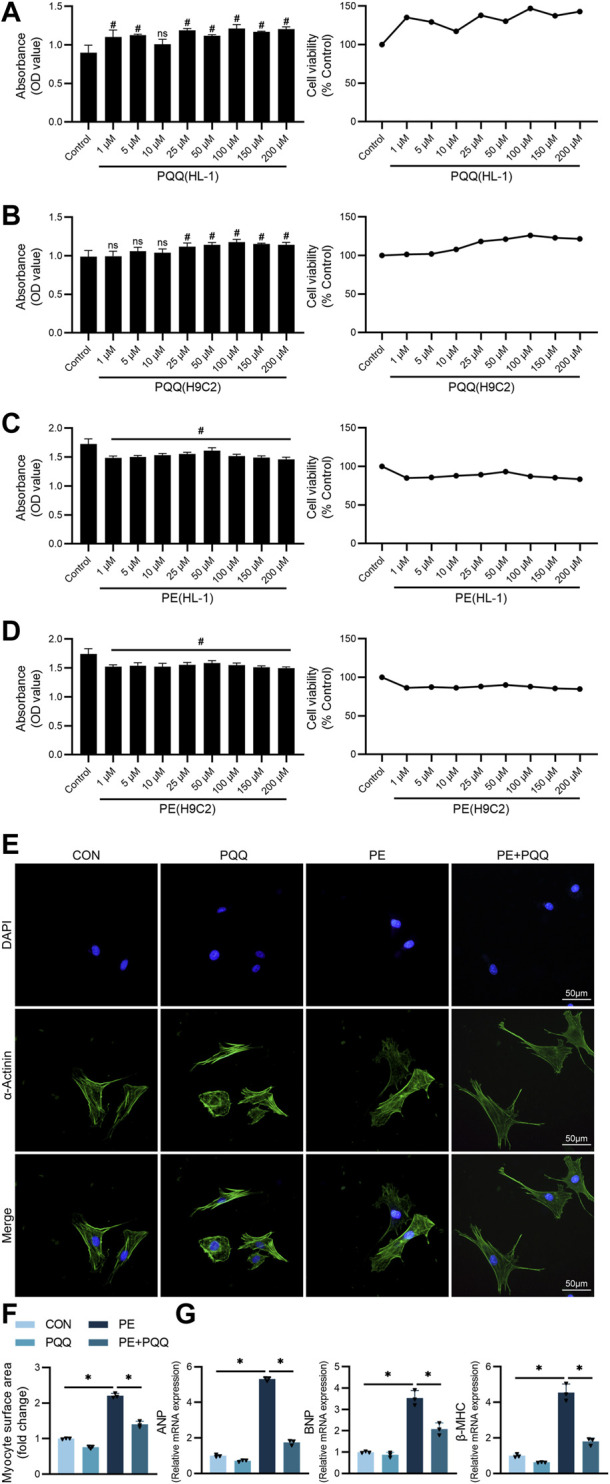
PQQ suppresses *in vitro* PE-induced cardiomyocyte hypertrophy. **(A)** Changes in HL-1 cell optical density values with increasing PQQ concentrations and the impact of different PQQ concentrations on HL-1 cell viability. **(B)** Changes in H9C2 cell optical density values with increasing PQQ concentrations and the impact of different PQQ concentrations on H9C2 cell viability. **(C)** Changes in HL-1 cell absorbance values with rising PE concentrations and the impact of different PE concentrations on HL-1 cell viability. **(D)** Changes in H9C2 cell absorbance values with rising PE concentrations and the impact of different PE concentrations on H9C2 cell viability. Control group HL-1 and H9C2 survivals were set to 100%. **(E–F)** Representative α-actinin staining images for primary cardiomyocytes with corresponding cell surface area quantification of cell surface area. **(G)** The relative expression of the hypertrophy biomarkers ANP, BNP, and β-MHC was assessed via qPCR. Data are means ± SEM. #*p* < 0.05 vs control; *p < 0.05 vs the PE group; ns, not significant. N = 3–4/group. Data were analyzed via one-way ANOVAs with Bonferroni post hoc tests.

To gain insight into the mechanisms whereby PQQ impacts cardiomyocytes in a hypertrophic context, an *in vitro* model system was next established. Briefly, HL-1 and H9C2 cells were treated for 24 h with a range of PQQ concentrations (1–200 μM), after which a CCK-8 assay was used to examine cell viability. As none of these doses were associated with cytotoxicity, a 100 μM PQQ dose was selected for subsequent use in light of prior studies (Xu et al., 2014) (Figures 3A,B). Using this same approach to examine the cytotoxic effects of PE treatment (Zhang et al., 2021), a 50 μM PE dose was selected for subsequent experimental use (Figures 3C,D).

Next, α-actinin staining was used to explore the ability of PQQ treatment to alter PE-associated changes in cardiomyocyte morphological characteristics consistent with hypertrophy. While untreated cardiomyocytes appeared filamentous and evenly distributed, PE treatment was associated with significant increases in cell size and increasingly irregular morphological characteristics, thus reaffirming the ability of PE to reliably induce cardiac hypertrophy. Relative to these PE-treated cells, however, cells treated with both PE and PQQ exhibited significant reductions in size and more regular cellular morphology, thus indicating that PQQ was able to significantly inhibit primary cardiomyocyte hypertrophy (Figures 3E,F). To further confirm these findings, the expression of the hypertrophy-associated biomarkers ANP, BNP, and β-MHC was assessed at the mRNA level in primary cardiomyocytes, revealing that all three of these genes were upregulated after 48 h in PE-treated cells, whereas PQQ pretreatment reversed this PE-induced upregulation of these genes (Figure 3G).

### PQQ suppresses the PE-driven induction of ferroptosis in cardiomyocytes

**FIGURE 4:**
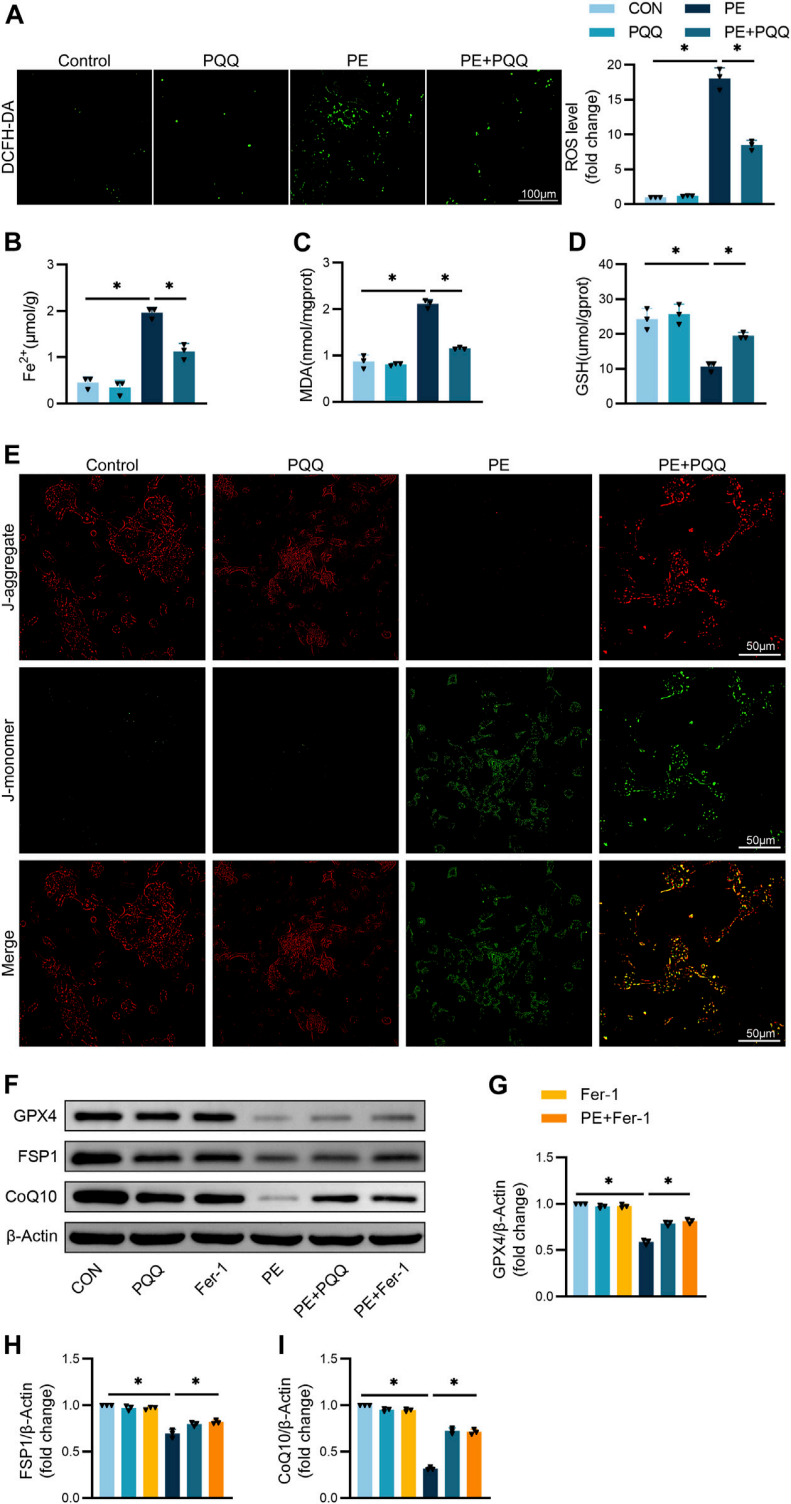
PQQ suppresses PE-induced *in vitro* cardiomyocyte ferroptotic death. **(A)** ROS levels in cardiomyocytes were quantified with a DCFH-DA fluorescent probe. **(B–D)** Iron, MDA, and GSH levels in murine cardiomyocytes were quantified with appropriate commercial kit. **(E)** Representative JC-1 green/red fluorescence images. **(F–I)** Representative Western immunoblots with corresponding quantification assessing GPX4, FSP1, and CoQ10 levels in primary cardiomyocytes. Data are means ± SEM. *p < 0.05. N = 3/group. Data were compared via one-way ANOVAs with Bonferroni post hoc tests.

To gain insight regarding the ability of PQQ to inhibit *in vitro* hypertrophic cardiomyocyte ferroptosis, DCFH-DA was next used to measure ROS levels in these cells. PE treatment for 48 h was associated with significantly elevated ROS production relative to the levels observed in the control or PQQ treatment groups, while PQQ effectively reversed PE-induced ROS production (Figure 4A). Further analyses of iron ion levels and the lipid peroxidation markers GSH and MDA revealed that Fe^2+^ and MDA levels were significantly higher following PE treatment relative to levels in control cells, while GSH levels were significantly reduced, and PQQ treatment significantly reversed all three of these PE-induced changes (Figures 4B–D).

JC-1 staining was further used to evaluate the impact of PQQ on the MMP of PE-treated primary cardiomyocytes, with monomeric (green) and polymeric (red) JC-1 being formed under conditions of normal MMP and hyperpolarization, respectively. More robust red fluorescent signal was observed for primary cardiomyocytes in the control and PQQ treatment groups, whereas PE treatment was associated with increased green signal that was reduced when cells were treated with both PE and PQQ. This suggested that PQQ can protect against the PE-induced disruption of MMP (Figure 4E).

We added Ferrostatin-1 (Fer-1), a specific inhibitor of ferroptosis, as a positive control in our experiments. Analyses of the ferroptosis-related Gpx4, FSP1, and CoQ10 protein levels further revealed that these three proteins were significantly upregulated in TAC-treated mice, but were reversed following PQQ administration, consistent with the results of adding the Fer-1 groups. These experiments revealed that PQQ is involved in the anti-ferroptotic activity (Figures 4F–I).

### PQQ exerts cardioprotective efficacy through a mechanism associated with YAP-related anti-ferroptotic activity

**FIGURE 5:**
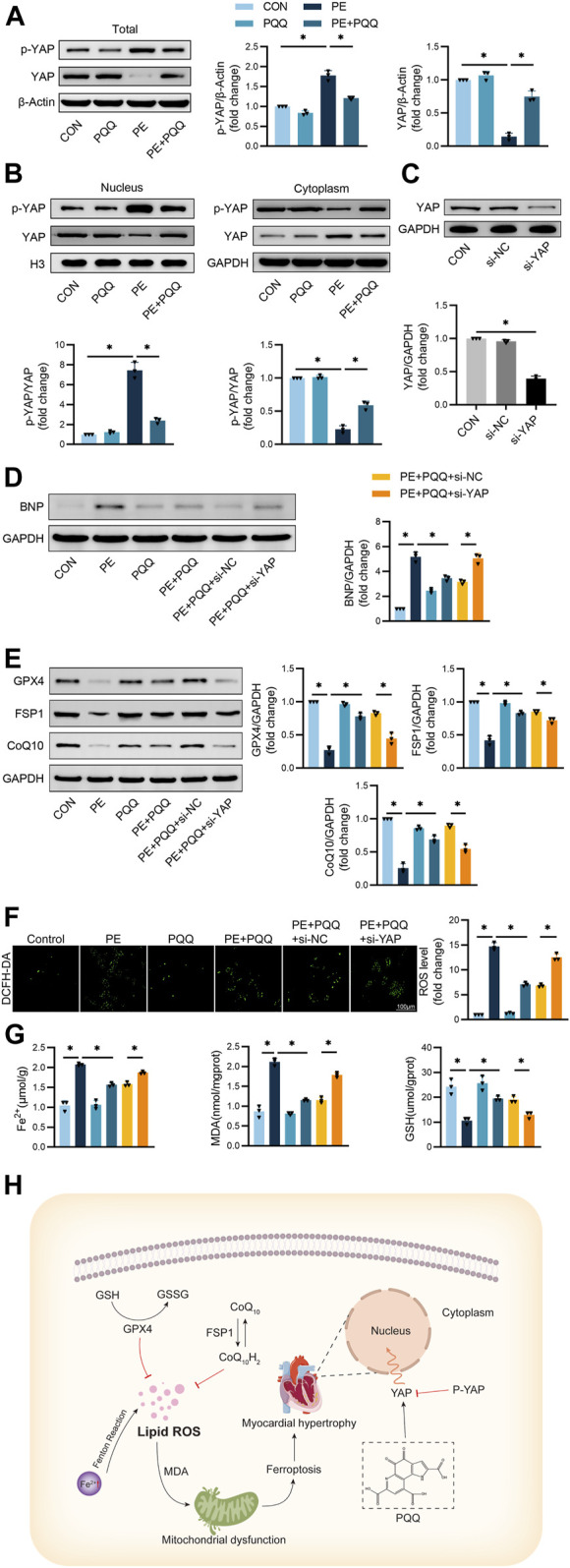
PQQ modulates YAP-related anti-ferroptotic activity to suppress myocardial hypertrophy **(A)** Representative Western immunoblots with corresponding quantification assessing total YAP and p-YAP levels in primary cardiomyocytes. **(B)** Representative Western immunoblots with corresponding quantification assessing nuclear and cytoplasmic YAP levels in primary cardiomyocytes. **(C)** Following si-NC or si-YAP transfection, Western immunoblotting was used to assess silencing efficacy in primary cardiomyocytes. **(D)** Representative Western immunoblots with corresponding quantification assessing BNP levels in primary cardiomyocytes. **(E)** Representative Western immunoblots with corresponding quantification assessing GPX4, FSP1, and CoQ10 levels in primary cardiomyocytes. **(F)** DCFH-DA fluorescence-based quantification of intracellular ROS levels in cardiomyocytes. **(G)** Iron, MDA, and GSH levels in murine cardiomyocytes were quantified with appropriate commercial kits. **(H)** Schematic overview of the inhibition of myocardial hypertrophy mediated by the PQQ-induced modulation of YAP-associated anti-ferroptotic activity. Data are means ± SEM. *p < 0.05. N = 3/group. Data were compared via one-way ANOVAs with Bonferroni post hoc tests.

Next, the relationship between the activity of PQQ and YAP signaling was assessed via Western immunoblotting. Significantly higher p-YAP levels and reduced total YAP levels were detected in PE-treated primary cardiomyocytes, while PQQ reversed these changes (Figure 5A), consistent with its ability to inhibit YAP phosphorylation. The PE group exhibited a significantly higher p-YAP/YAP ratio as compared to the control group, whereas the opposite outcome was observed in the cytoplasm. PQQ treatment, however, significantly reduced this p-YAP/YAP ratio, thus confirming that YAP phosphorylation is induced in hypertrophic cardiomyocytes such that YAP nuclear translocation is inhibited, whereas PQQ treatment can reverse this change (Figure 5B).

To verify the functional role that YAP plays as a regulatory mediator of the anti-ferroptotic effects of PQQ treatment, YAP was next knocked down in cardiomyocytes prior to treatment with PE. Western immunoblotting was used to confirm the efficiency of YAP knockdown in cells transfected with si-YAP or si-NC constructs (Figure 5C). Levels of the cardiomyocyte hypertrophy marker BNP were significantly increased in PE-treated cells, while PQQ treatment failed to fully reverse this change in si-YAP transfected cardiomyocytes (Figure 5D). PE-treated cells additionally exhibited lower levels of the ferroptosis-associated proteins GPX4, FSP1, and CoQ10 relative to control cells, while these levels were significantly increased in the PE + PQQ group relative to the PQQ treatment group. YAP silencing, however, interfered with the ability of PQQ to support the upregulation of these proteins (Figure 5E). Knocking down YAP effectively prevented PQQ-mediated reductions in ROS levels in the PE + PQQ + si-YAP treatment group (Figure 5F). Meanwhile, we again demonstrated that YAP correlated with ferroptosis by measuring Fe^2+^ concentration, GSH and MDA levels, and knockdown of YAP significantly attenuated the anti-ferroptosis ability of PQQ (Figure 5G). Together, these data indicated that PQQ holds promise as a cardioprotective agent that prevents myocardial hypertrophy at least in part via YAP-related anti-ferroptotic mechanisms (Figure 5H).

## Discussion

Pathological myocardial hypertrophy is characterized by the death of myocardial cells and concomitant fibrosis that ultimately leads to reductions in systolic and diastolic function, contributing to the onset and progression of heart failure. Aggressively treating myocardial hypertrophy is thus critical as a means of preventing these adverse outcomes. Here, PQQ administration to TAC-operated mice was found to improve cardiac remodeling, dysfunction, and fibrosis, with similar results being confirmed *in vitro* using PQQ-treated cardiomyocytes that had been exposed to PE to model hypertrophy. Further research revealed that YAP-related anti-ferroptotic activity was tied to the cardioprotective effects of PQQ, with YAP inhibition representing an effective means of partially reversing the cardioprotective and anti-ferroptotic effects of PQQ treatment.

Cell death is an essential hallmark of life, and ferroptosis is a specific form of cell death distinct from necrosis, apoptosis, or autophagy that is linked to certain physiological and pathological processes. The induction of ferroptosis is tied to lipid peroxidation, amino acid metabolism, and iron metabolism, and has been linked to conditions including myocardial ischemia/reperfusion (MI/R), cardiac hypertrophy, cardiomyopathy, atherosclerosis, heart failure, and abdominal aortic aneurysm. Among other things, it is mentioned that the occurrence of ferroptosis can exacerbate myocardial hypertrophy (Zhang et al., 2022). Excessively high iron ion levels within cells can lead to hydroxyl radical production via the Fenton reaction through interactions with hydrogen peroxide, thus promoting ROS production, lipid peroxidation, and the generation of oxidative byproducts such as MDA that are highly toxic, contributing to ferroptosis (Zeng et al., 2019). These free iron ions can also enter mitochondria, spurring oxidative stress induction therein while impairing their function such that mitochondrial ROS production increases while mitochondrial respiration is impaired, with concomitant mitochondrial swelling and MMP depolarization (Wang et al., 2020). With respect to amino acid metabolism, cell metabolism-related lipid hydrogen peroxide is reduced by Gpx4 to yield lipid alcohols, thus shielding cells from oxidative stress-associated damage. However, GSH depletion and reduced Gpx4 activity in the context of ferroptosis interfere with this pathway such that lipid oxidates interact with Fe^2+^, generating large lipid peroxide (LPO) volumes (Friedmann Angeli et al., 2014). Ferroptosis is also regulated by the FSP1-CoQ10 axis, with FSP1 serving as a CoQ1 oxidoreductase. CoQ10 undergoes N-terminal actinylation as a lipid modification that promotes the targeting of FSP1 to the plasma membrane, thus facilitating coenzyme Q reduction to inhibit its activity (Bersuker et al., 2019; Tang et al., 2021), with CoQ10 reduction additionally mitigating oxidative stress, preventing lipid ROS accumulation, suppressing adipocyte differentiation (Xu et al., 2017), and inhibiting phospholipid peroxidation and ferroptotic death (Santoro, 2020). In line with these previous results, TAC-induced cardiac samples exhibited elevated MDA and Fe^2+^ levels together with higher levels of ROS production, mitochondrial phenotypes consistent with ferroptosis including decreased cristae and a smaller volume, and reductions in the expression of GPX4, GSH, FSP1, and CoQ10. Similar results were also observed for PE-treated primary cardiomyocytes. Together, these results thus highlight a close link between myocardial hypertrophy and ferroptosis such that inhibiting this form of cell death can slow or prevent the onset of pathological myocardial hypertrophy.

PQQ is a B vitamin-related water-soluble, thermally stable redox coenzyme with a relative molecular mass of 330.2 and a molecular formula of C_14_H_6_N_2_O_8_ that is highly abundant in bacteria, plants, and animals wherein it can be found in both reduced and oxidized forms (Kumazawa et al., 1995). Therefore, these series of properties make PQQ more valuable for research than other compounds. PQQ has previously been shown to exert cardioprotective activity in the context of myocardial infarction (Zhu et al., 2004), diabetic cardiomyopathy (Qu et al., 2022), lipid abnormalities (Bauerly et al., 2011), and COVID-19 mRNA vaccine-related cardiac inflammation (Boretti, 2022). Whether PQQ can alleviate pathological myocardial hypertrophy via anti-ferroptotic mechanisms, however, has not been previously assessed. Accordingly, the present study utilized a pressure overload-induced murine model of *in vivo* cardiac hypertrophy together with PE-treated primary hypertrophic cardiomyocytes to explore the ability of PQQ to modulate ferroptotic induction. Overall, PQQ was found to promote cell survival through antioxidant activities as demonstrated in iron ion, MDA, and DCFH-DA staining assays. PQQ also increased average MMP values in cardiomyocytes and alleviated hypertrophy-associated changes in mitochondrial morphology consistent with its ability to restore mitochondrial homeostasis. Consistently, PQQ was able to increase GSH expression and Gpx4 activity in cardiomyocytes *in vivo* and *in vitro,* in addition to augmenting the FSP1-CoQ10 axis to protect against ferroptosis-associated damage.

As a critical transcriptional transactivator and key effector component of the Hippo pathway, YAP regulates the expression of a diverse array of cell growth-related genes (Gumbiner and Kim, 2014) and has been linked to the pathogenesis of pulmonary hypertension (Bertero et al., 2016), atherosclerosis (Wang et al., 2016), and other vascular diseases. The phosphorylation of YAP enables it to bind to 13-3-3, thus preventing it from translocating into the nucleus. Dephosphorylated YAP undergoes nuclear inactivation (Schlegelmilch et al., 2011). Prior studies have not explored the link between YAP and the regulation of ferroptotic cell death within hypertrophic cardiomyocytes. There is evidence that melatonin can promote YAP upregulation in cardiomyocytes, thereby mitigating mitochondrial oxidative damage and ferroptotic activity induced in response to doxorubicin (Sun et al., 2022). Separately, Niu et al. (Niu et al., 2021) determined that Echinatin can regulate the Hippo/YAP signaling axis to protect against MI/R injury. In this study, YAP was found to be significantly downregulated in both a murine model of TAC-induced cardiomyocyte hypertrophy and in PE-treated primary cardiomyocytes with a corresponding increase in p-YAP levels, whereas PQQ treatment partially reversed these phenotypes. These data strongly suggest that cardiomyocyte hypertrophy drives the phosphorylation of YAP and prevents it from translocating to the nucleus, while PQQ interferes with this process and thereby enables YAP nuclear translocation. To gain further insight regarding the link between the expression of YAP and ferroptotic cell death, YAP was knocked down *in vitro* using a siRNA construct. YAP silencing resulted in the downregulation of the ferroptosis-related proteins GPX4, FSP1, and CoQ10, thus supporting a role for YAP as an anti-ferroptotic mediator. Thus, despite YAP’s effects on gene transcription, mitochondrial function and oxidative stress are known, the role of PQQ in blunting PE-induced YAP phosphorylation is where the novelty of this study lies. Meanwhile, according to previously published work (Yang and Chi, 2020), triggering ferroptosis is beneficial for the treatment of YAP-activated cancers such as kidney, ovarian and breast cancers. In contrast, our study showed that YAP was inhibitory to ferroptosis during the development of normal cardiomyocytes into pathologically hypertrophied cardiomyocytes. This may be related to the different cell types and possibly a dynamically altered process which needs further research. Overall these data thus revealed that PQQ can serve as a cardioprotective antioxidant capable of suppressing cardiomyocyte hypertrophy *in vitro* and *in vivo* in part via inhibiting ferroptotic cell death through the modulation of the YAP signaling axis.

There are some limitations to this study. At first, based on the previous study, we only evaluated mice at 6 weeks after TAC surgery and lacked observations at earlier time points. Secondly, the direct mechanism of action and targets against YAP signaling were not further validated in vivo experiments. Furthermore, we used HL-1 and H9C2 cells for indirect measurement of cellular activity, while further studies on direct assay of primary cardiomyocyte activity are still needed to be explored. In addition, PQQ may also protect against pathological myocardial hypertrophy via other signaling pathways, highlighting the need for further research aimed at clarifying the underlying mechanisms.

## Conclusion

In summary, these data indicate that PQQ can modulate YAP, thereby inhibiting the induction of cardiomyocyte ferroptosis to protect against myocardial hypertrophy. These results provide a valuable foundation for future efforts to utilize PQQ as a cardioprotective agent or a basis for further drug development efforts aimed at preventing long-term cardiogenic heart failure. In addition, YAP offers great promise as a target for therapeutic intervention and potentially as a biomarker linked to the progression of pathological myocardial hypertrophy.

## Data Availability

The original contributions presented in the study are included in the article/Supplementary Materials, further inquiries can be directed to the corresponding author.
